# Mental health and associated factors among undergraduate students during Covid-19 pandemic in Chile

**DOI:** 10.1192/j.eurpsy.2022.861

**Published:** 2022-09-01

**Authors:** S. Ramirez, J. Valdés, F. Díaz, F. Solorza, P. Christiansen, G. Lorca, J. Gaete

**Affiliations:** 1 Universidad de los Andes, Faculty Of Education, Santiago, Chile; 2 Universidad de los Andes, School Of Medicine, Santiago, Chile

**Keywords:** mental health, Undergraduate students, substance use, Covid-19

## Abstract

**Introduction:**

Very few studies have explored mental health among undergraduate students in Chile, especially during the Covid-19 pandemic. International studies have estimated the prevalence of depression at around 28%.

**Objectives:**

i) To determine the prevalence of mental health problems among undergraduate students at a private university in Chile; ii) to explore the associated factors in the context of the Covid-19 pandemic.

**Methods:**

This was a cross-sectional study, approved by the Ethical Committee of the Universidad de los Andes, Santiago Chile (CEC201984). Undergraduate students completed an online survey between August and September 2020. Mental health was assessed using The Depression, Anxiety and Stress Scale-21; suicidality, using the Columbia Suicide Severity Rating Scale; insomnia using the Insomnia Severity Index; and several individual, family, and university factors. Variables regarding the Covid-19 were also assessed, such as personal and family history of covid-19 contagion and death of family members due to Covid-19. A multivariate logistic analysis was performed.

**Results:**

5,037 students responded to the survey. 70.4% were female, mean age, 21 years. 37.1% had depression; 38%, anxiety; 54.6%, stress; 32.6%, insomnia; and 20.5%, suicidal ideation (last month). The most important risk factors were non-medical use of benzodiazepine and fear of contracting Covid-19; the most relevant protective factors were high family functionality and a high sense of university belonging.

**Conclusions:**

This is one of the first studies that has evaluated mental health among undergraduate students in the context of the Covid-19 pandemic in Chile. The findings showed concerning levels of mental health problems.

**Disclosure:**

No significant relationships.

